# Chronic obstructive pulmonary disease effect on the prevalence and postoperative outcome of abdominal aortic aneurysms: A meta-analysis

**DOI:** 10.1038/srep25003

**Published:** 2016-04-26

**Authors:** Jiang Xiong, Zhongyin Wu, Chen Chen, Wei Guo

**Affiliations:** 1 Department of Vascular and Endovascular Surgery, Chinese PLA General Hospital, Beijing, P.R. China; 2 Department of General Surgery, Affiliated Hospital of Chengde Medical College, Chengde, Hebei, P.R. China; 3 Department of Health Policy and Management, Jiann-Ping Hsu College of Public Health, Georgia Southern University, Statesboro, GA, USA

## Abstract

Epidemiologic evidence suggested chronic obstructive pulmonary disease (COPD) might increase risk for abdominal aortic aneurysm (AAA). However, the association between COPD and AAA remains inconclusive. We searched PubMed and Cochrane databases until June 2015. Forty-eight articles were included for meta-analysis. COPD was found to be positively associated with AAA, regardless of study design and smoking status. AAA mortality is higher among COPD patients compared with non-COPD patients (postoperative [adjusted OR 2.11; 95% CI 1.33–3.34]; long-term [adjusted OR 1.70; 95% CI 1.37–2.12]). But the association between postoperative mortality and COPD was not found to be significant in patients underwent endovascular aneurysm repair (mixed OR 2.53; 95% CI 0.70–9.18). Rupture AAA may increase the postoperative mortality in COPD patients (rupture [adjusted OR 4.75; 95% CI 2.07–10.89]; non-rupture [adjusted OR 1.97; 95% CI 1.11–3.49]). The AAA postoperative morbidity was found to be positively associated with COPD (adjusted OR 1.59; 95% CI 1.14–2.21). Increased COPD severity may increase the long-term mortality (medical versus oxygen dependent: [OR 1.26; 95% CI 1.07–1.49] versus [OR 2.79; 95% CI 2.24–3.49]). In conclusion, COPD may increase the risk of AAA, morbidity and mortality of AAA patients underwent endovascular aortic repair.

Epidemiological data revealed that chronic obstructive pulmonary disease (COPD) affects 34–200 out of 1000 patients older than 65 years^1^. According to the Global Burden of Disease (GBD) 2010, COPD affects approximately 329 million people (4.8% of the population) in the world as of 2010^2^, and is the fourth leading cause of morbidity and mortality in the United States^3^. Some population-based screenings (PBS) indicated that the prevalence of abdominal aortic aneurysm (AAA) among COPD patients was 4% to 6%^4^,^5^,^6^,^7^,^8^. Other studies found even higher prevalence varied from 7% to 11%^9^,^10^,^11^. The above evidence suggested that COPD might be a risk factor for AAA. Smoking and increased elastolytic activity in lung and aorta are found to be strongly associated with both COPD and AAA^10^,^12^,^13^,^14^,^15^,^16^. The causal relationship between smoking and COPD has been reported in previous studies; however, smoking was only mentioned as a risk factor of AAA. The precise mechanism about how COPD can influence the development and prognosis of AAA remains to be unclear. Previous studies revealed possible association between COPD and AAA^4^,^6^,^17^,^18^ adjusting for smoking or not. To our knowledge no study up to date has focused on the comprehensive and systematic analysis of this issue and no conclusion has been drawn. Moreover, there is also limited data on AAA postoperative clinical outcome among COPD patients. To better understand this issue, we performed a meta-analysis to investigate the influence of COPD on prevalence of AAA, as well as the AAA postoperative mortality and morbidity among COPD patients.

## Methods

### Data Sources and Search Strategy

PubMed and Cochrane databases were searched to identify studies published from January 1982 to June 2015 (ultrasound technology was not available before 1982^19^). A combination of the following search terms was used: “abdominal aortic aneurysm”, “chronic obstructive pulmonary disease”, “chronic obstructive lung disease”, “chronic obstructive airway disease” and “emphysema”. We restricted our research to human studies and published in English language. The review articles were initially involved for identifying potential eligible data, and were not included for meta-analysis. Two authors independently reviewed each potential study for eligibility. The reference list of eligible studies was screened to further identify relevant studies by a manual search. Type of study design was not restricted.

### Inclusion Criteria

Studies were included if they: (1) reported odd ratio (OR), relative risk (RR) or hazard rate (HR) of AAA, or if the original data was available for calculating prevalence or incidence of AAA (i.e., for assessing prevalence of AAA in COPD patients); (2) focused on infrarenal AAA patients who received endovascular aneurysm repair (EVAR) or open repair (i.e., for examination of AAA postoperative outcomes among COPD patients).

### Exclusion Criteria

Studies were excluded if they reported on suprarenal AAA or thoracoabdominal aneurysm, or thoracoabdominal approaches. Additionally, the following were excluded: nonhuman studies, studies published prior to 1982, studies with data duplicated, editorials, case reports and letters.

### Data Extraction and Quality Assessment

The following data were independently extracted by two researchers (J.X., Z.W.): study design, AAA screening method, country of origin, age, gender, intervention strategy, duration of follow-up, number of defined AAA and COPD patients, outcomes (e.g., 30-day mortality, long-term mortality, 30-day morbidity, long-term morbidity), and type of analysis (univariate or multivariate calculations). Discrepancies in data extraction were resolved through discussion. Study quality was quantified based on the Newcastle-Ottawa Scale^20^. According to this assessment scale, all the selected cohort and case-control studies were evaluated on three groups of items, including: (1) the selection of study; (2) the comparability of the groups; and (3) the ascertainment of outcomes of interest. The scale uses a star system for assessment. Stars were awarded for each quality item, with a maximum of 9 stars. Studies with five stars or more were considered to be “good” in quality. Studies with four stars or less were considered to be “fair” in quality.

### Statistical Analysis

Studies were separately analyzed according to whether they reported on prevalence/incidence or clinical outcomes using RevMan v.5.0 (The Cochrance Collaboration, http://ims.cochrane.org/revman) and STATA 12 software (StataCorp, College Station, Texas, USA). The overall estimates, such as RR, HR or OR with corresponding 95% confidence intervals (CIs), were combined in a meta-analysis using the generic inverse variance option in RevMan to obtain a pooled result. Because the prevalence of AAA in COPD patients was low (less than 10%), we assumed similarity between the OR, RR and HR. The adjusted estimates of risk were employed when available. When data had been presented in original form, we input total and number of events into RevMan 5.0 for calculating estimates and then used the generic inverse variance method. The meta-analysis was performed using the Mantel-Haenszel method. We used I^2^ values and χ^2^ statistic to assess heterogeneity. If I^2^ > 50% significant heterogeneity between-study were indicated, and random-effects models were used to estimate pooled effect sizes; otherwise, fixed-effect models were applied. All pooled estimates of risk were reported with 95% CIs. If meta-analysis included 10 studies or more, potential publication bias was evaluated by using the Egger test, and the results were represented graphically with Begg funnel plots of the natural log of the OR versus its standard error.

### Sensitivity Analysis

Prespecified sensitivity analysis was conducted for studies reporting different treatment (open repair or EVAR), different hemodynamic status (ruptured or non-ruptured) and different grades of COPD (untreated, medical or oxygen dependent) where they were available.

## Results

**Figure 1:**
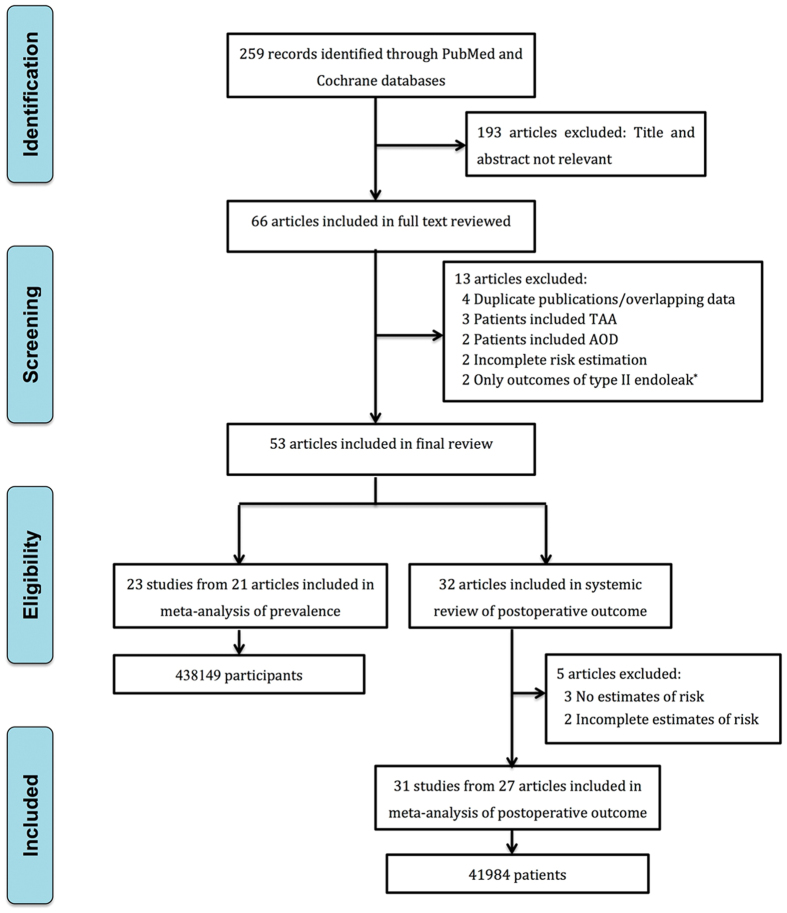
Flow chart of articles included in the meta-analysis. TAA: thoracic aortic aneurysm; AOD: aortic occlusive disease. ^*^The type II endoleak was not considered as the complication.

A total of 259 records were identified by the initial search strategy (Fig. 1). After screening the titles and abstracts, 66 articles were selected to be further reviewed. Among those 66 articles, 13 articles were excluded (Fig. 1). Of the selected 53 articles, 21 reported on the prevalence of AAA in COPD patients and included 438149 participants, and 32 articles provided data on clinical outcomes of endovascular aortic repair (EVAR) or open surgery among COPD patients. Majority included articles (n = 43) were ranked as “good” in methodological quality, and the other ten articles were considered to have fair methodological quality (Online Table 1).

### Prevalence of AAA in COPD

**Figure 2:**
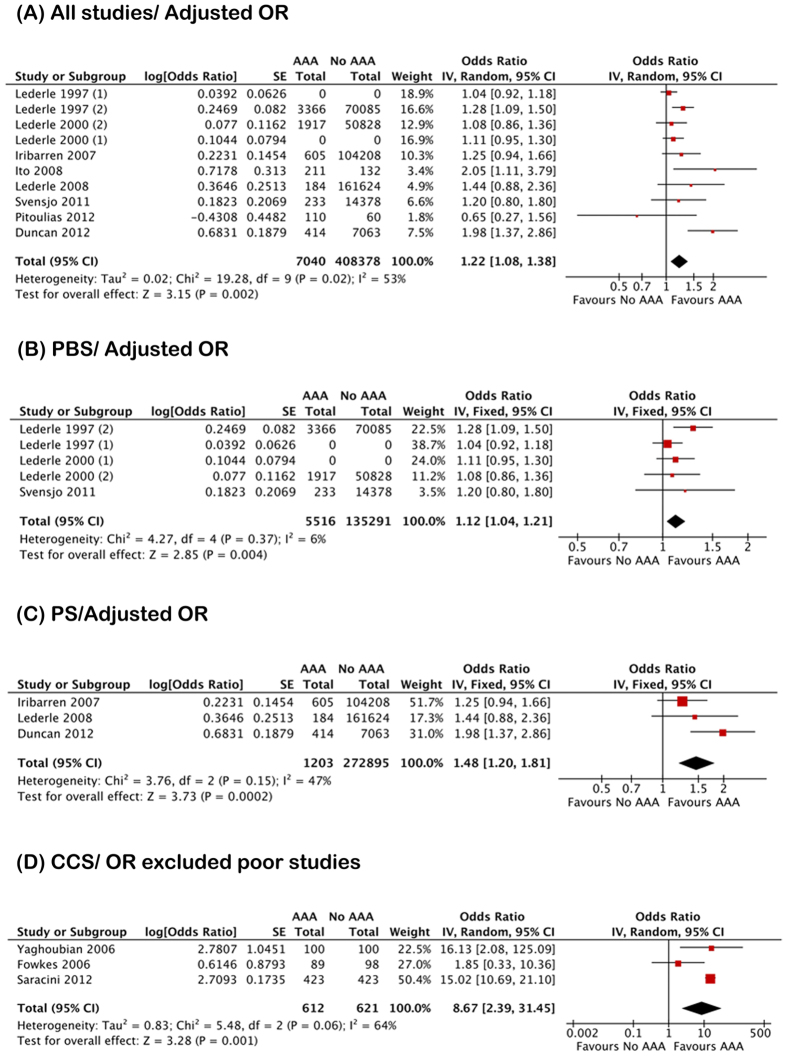
The association between chronic obstructive pulmonary disease and abdominal aortic aneurysm: Pooled adjusted odd ratio. (**A**) All studies. (**B**) Population based screenings. (**C**) Prospective studies. (**D**) Case-control studies excluded poor studies.

Twenty-three studies from 21 articles reported the prevalence of AAA in COPD. According to the categorization of studies based on AAA diameter (3.0–3.9 cm vs. ≥4.0 cm), four studies were extracted from two of Lederle’s articles. The main characteristics of the 23 studies were listed in Table 1. Based on those 23 studies (AAA/Non-AAA: 8869/429280), when combining the adjusted and unadjusted data, the pooled estimates indicated a significant positive association between COPD and the prevalence of AAA (OR 1.78; 95% CI 1.35–2.35; I^2^ 93%) (Online Fig. 1A). The possibility of publication bias was low (P = 0.143) (Online Fig. 2A). Based on the ten studies which reported adjusted OR (AAA/Non-AAA: 7040/408378), a pooled adjusted estimate indicated a slight but significant positive association between COPD and prevalence of AAA (OR 1.22; 95% CI 1.08–1.38; I^2^ 53%) (Fig. 2A). The possibility of publication bias was low (P = 0.193) (Online Fig. 2B).

According to the study type, AAA prevalence data were separately extracted from 11 PBS, three prospective studies (PS) and 9 case-control studies (CCS). If adjusted data were employed when available, the pooled analysis from the 11 PBS (AAA/Non-AAA: 6450/154414) revealed a significant positive association between COPD and AAA (OR 1.56; 95% CI 1.21–2.00; I^2^ 89%) (Online Fig.1B). The possibility of publication bias was low (P = 0.151) (Online Fig. 2C). Based on five PBS with adjusted estimates (AAA/Non-AAA: 5516/135291), the pooled result indicated a slight positive association between COPD and the prevalence of AAA (OR 1.12; 95% CI 1.04–1.21; I^2^ 6%) (Fig. 2B). A strong positive association (OR 1.48; 95% CI 1.20–1.81; I^2^ 47%) was found in the pooled adjusted estimates based on 3 PS (AAA/No AAA: 1203/272895) (Fig. 2C). However, the pooled result from the 9 CCS (AAA/No AAA: 1216/1971) demonstrated no significant association between COPD and AAA (OR 2.27; 95% CI 0.90–5.76; I^2^ 93%) (Online Fig.1C). After excluding 6 studies with poor control group (5 studies with arteriosclerosis and 1 study with TAA) from the analysis, the pooled result based on the remaining 3 studies (AAA/Non-AAA: 612/621) suggested a strongly positive association between COPD and AAA (OR 8.66; 95% CI 2.39–31.44; I^2^ 64%) (Fig. 2D).

**Figure 3:**
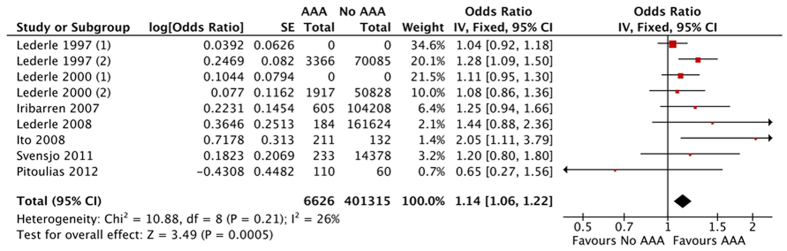
The association between abdominal aortic aneurysm and chronic obstructive pulmonary disease: Pooled odd ratio adjusted smoking status.

Nine studies (AAA/Non-AAA: 6626/401315) reported data adjusted for smoking status. The pooled result showed that COPD was positive associated with AAA with adjustment of smoking status (OR 1.14, 95% CI 1.06–1.22, I^2^ 26%) (Fig. 3).

### Clinical outcomes in AAA patients with COPD

Twenty-seven articles were included for estimation of postoperative clinical outcome of AAA in COPD patients. Based on the purpose of this meta-analysis, we identified 9 studies from 4 of the articles according to disease characteristics and type of surgery. Finally, 31 studies from 27 articles were identified for meta-analysis of postoperative outcome, and a total of 41984 patients were included (Table 2).

### 30-day/in-hospital mortality

**Figure 4:**
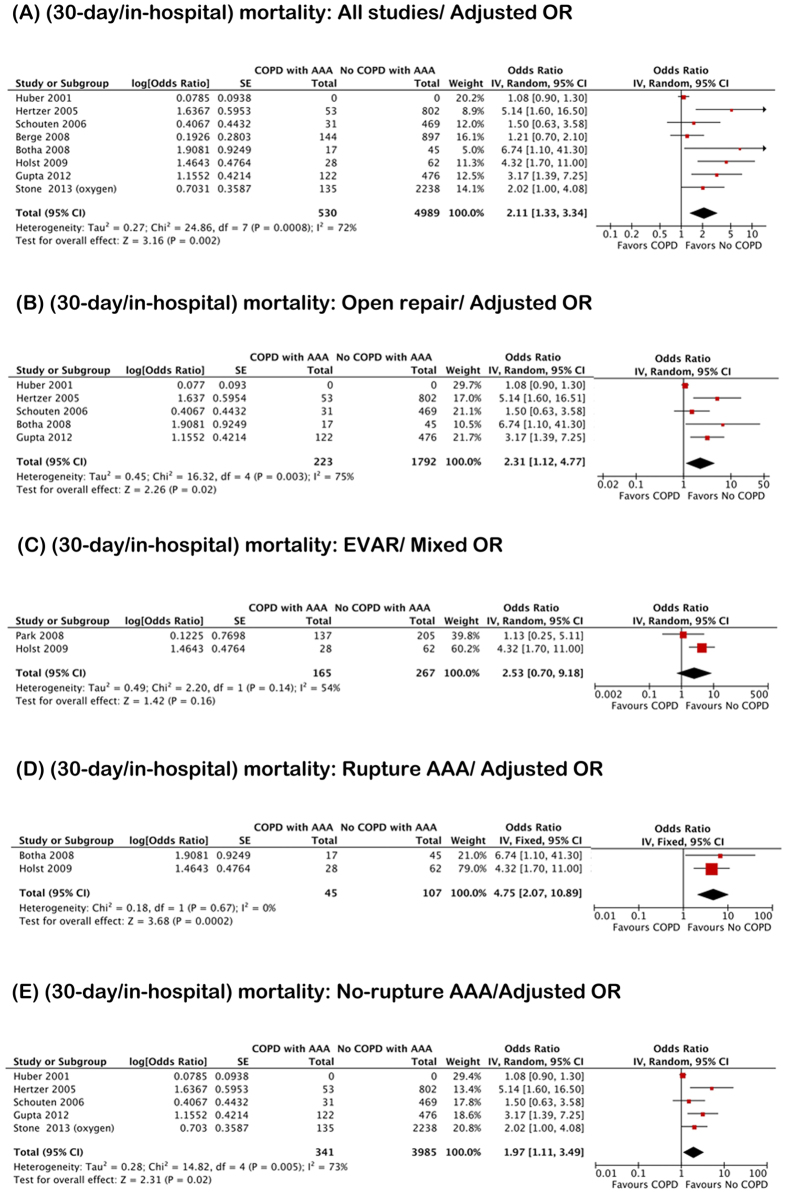
Cumulative operative mortality in abdominal aortic aneurysm (AAA) patients with chronic obstructive pulmonary disease: Pooled adjusted/mixed odd ratio. (**A**) All studies. (**B**) Studies with AAA accepted open repair. (**C**) Studies with AAA accepted endovascular aneurysm repair. (**D**) Studies with rupture AAA. (**E**) Studies with no rupture AAA.

Eighteen studies (COPD/Non-COPD: 3493/14150) reported 30-day/in-hospital mortality of AAA after repair. The in-hospital mortality was found in 6 studies and 30-day mortality in 12 studies. When combining adjusted and unadjusted data, the pooled result suggested that COPD was positively associated with 30-day/in-hospital mortality of AAA after repair (OR 1.58; 95% CI 1.22–2.03; I^2^ 60%) (Online Fig. 3A). The possibility of publication bias was high (P = 0.01). (Online Fig. 2D). If analysis was restricted to studies providing adjusted data (COPD/No-COPD: 530/4989), pooled analysis showed an increased 30-day/in-hospital mortality in COPD patients compared with non-COPD patients (OR 2.11; 95% CI 1.33–3.34; I^2^ 72%) (Fig. 4A).

To evaluate the treatment effect on AAA clinic outcome, the studies were divided into two subgroups according to type of treatment (open repair vs. EVAR). The data for estimating 30-day/in-hospital mortality of AAA after open repair was identified from 13 studies (COPD/Non-COPD: 2955/10801). The pooled result (including both adjusted and unadjusted data) suggested that COPD was strongly and positively associated with the 30-day/in-hospital mortality of AAA after open repair (OR 1.43; 95% CI 1.08–1.90; I^2^ 62%) (Online Fig. 3B). The possibility of publication bias was low (P = 0.057) (Online Fig. 2E). If analysis was restricted to the 5 studies with adjusted data (COPD/Non-COPD: 223/1792), pooled analysis showed an increased 30-day/in-hospital mortality rate in COPD patients compared with non-COPD patients (OR 2.31; 95% CI 1.12–4.77; I^2^ 75%) (Fig. 4B). Two studies (COPD/Non-COPD: 165/267) provided data for estimating the 30-day/in-hospital mortality of AAA after EVAR. The pooled result revealed no significant association between COPD and the 30-day/in-hospital mortality of AAA after EVAR (OR 2.53; 95% CI 0.70–9.18; I^2^ 54%) (Fig. 4C). The above results suggested that COPD increased postoperative mortality of AAA patients with the receipt of open repair but not EVAR.

To evaluate the effect of AAA types on the clinical outcomes, the studies were divided into two subgroups (rupture AAA vs. non-rupture AAA). The data for estimating the postoperative 30-day/in-hospital mortality for patients with rupture AAA was identified from 6 studies (COPD/No-COPD: 152/342). The mixed pooled result suggested that COPD was positively associated with postoperative 30-day/in-hospital mortality of rupture AAA patients (OR 2.72; 95% CI 1.14–6.48; I^2^ 69%) (Online Fig.3C). If analysis was restricted to studies providing adjusted data (COPD/Non-COPD: 45/107), the pooled analysis showed that the postoperative 30-day/in-hospital mortality of rupture AAA patients was strongly positive associated with COPD status (OR 4.75; 95% CI 2.07–10.89; I^2^ 0%) (Fig. 4D). Ten studies (COPD/Non-COPD: 1405/6518) provided data for estimating 30-day/in-hospital mortality for non-rupture AAA patients after receiving elective repair. The mixed pooled result suggested that COPD was slightly and positively associated with the 30-day/in-hospital mortality of non-rupture AAA after elective repair (OR 1.23; 95% CI 1.05–1.44; I^2^ 50%) (Online Fig. 3D). The possibility of publication bias was low (P = 0.162) (Online Fig. 2F). When the unadjusted data were removed from the analysis, the pooled adjusted data (COPD/No-COPD: 341/3985) suggested the postoperative 30-day/in-hospital mortality of non-rupture AAA patients was slightly positive associated with COPD status (OR 1.97; 95% CI 1.11–3.49; I^2^ 73%) (Fig. 4E).

### 30-day/in-hospital morbidity

**Figure 5:**
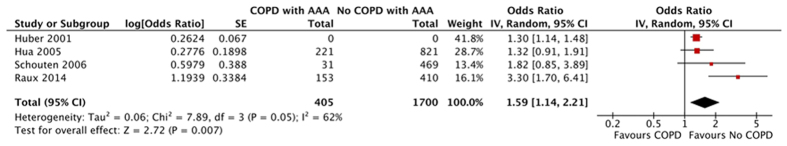
Cumulative operative morbidity in abdominal aortic aneurysm patients with chronic obstructive pulmonary disease: Pooled adjusted odd ratio.

Eight studies (COPD/Non-COPD: 712/2499) were included for meta-analysis regarding 30-day/in hospital morbidity among AAA patients. The mixed pooled result suggested that COPD was positively associated with 30-day/in-hospital morbidity of AAA receiving repair surgery (OR1.37; 95% CI 1.23–1.54; I^2^ 33%) (Online Fig. 4). Pooled estimates based on adjusted data (COPD/No-COPD: 405/1700) showed similar odds of getting complications among COPD patients and non-COPD patients (OR 1.59; 95% CI 1.14–2.21; I^2^ 62%) (Fig. 5).

### Long-term mortality

**Figure 6:**
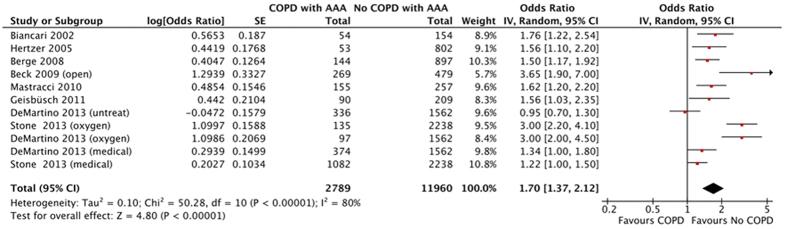
Cumulative long-term mortality in abdominal aortic aneurysm patients with chronic obstructive pulmonary disease: Pooled adjusted odd ratio.

Thirteen studies (COPD/Non-COPD: 2917/12243) were included for meta-analysis regarding AAA long-term mortality. The definition of long-term varied from 1 year to 20 years. The mixed pooled estimate suggested that COPD was positively associated with long-term postoperative mortality of AAA patients (OR1.59; 95% CI 1.26–2.01; I^2^ 84%) (Online Fig. 5). The possibility of publication bias was low (P = 0.47). (Online Fig. 2G). After removing studies with unadjusted data, the pooled adjusted estimate (COPD/No-COPD: 2789/11960) indicated higher AAA long-term mortality in COPD patients compared with non-COPD patients (OR 1.70; 95% CI 1.37–2.12; I^2^ 80%) (Fig. 6). The possibility of publication bias was low (P=0.061) (Online Fig. 2H).

**Figure 7:**
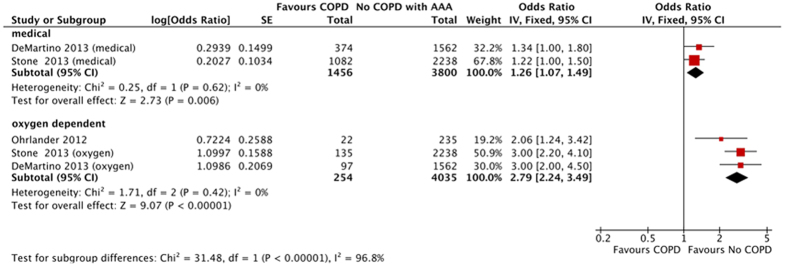
Pooled result of long-term mortality in abdominal aortic aneurysm patients with chronic obstructive pulmonary disease: Medical treatment vs. Oxygen dependent treatment.

Five studies were included for meta-analysis focusing on the influence of COPD grade (medical and oxygen dependent) on AAA long-term mortality. The pooled results showed that COPD is associated with increased AAA long-term mortality regardless of COPD grade (medical COPD: OR 1.26; 95% CI 1.07–1.49; I^2^ 0%; oxygen dependent COPD: OR 2.79; 95% CI 2.24–3.49; I^2^ 0%) (Fig. 7).

### Long-term morbidity

Two studies reported long-term postoperative morbidity among AAA patients. In a prospective, multicenter clinical trail of EVAR (923 patients), COPD was not found to be associated with the probability of AAA rupture (P = 0.98) and the probability of conversion events (P = 0.18) during a 5-year follow up period^21^. However, EUROSTAR collaborators registry published the result of conversion from 1871 enrolled patients who underwent EVAR, and supported that COPD was significantly and positively associated with the increased risk of conversion in a 1.5-year follow up period^22^.

## Discussion

According to our pooled mixed data and pooled adjusted data, we found that patients with COPD had 1.22 (pooled adjusted OR) to 1.78 (pooled mixed OR)-fold increased risk of AAA presence than those without COPD. When categorized by type of study, the pooled result showed that COPD was significantly and positively associated with AAA in PBS and PS studies, but CCS indicated no significant association. After excluding 6 studies with poor control group (5 studies with arteriosclerosis and 1 study with TAA) in CCS, the pooled result was found to be consistent with that were found in PBS and PS studies. As a result, in general, COPD was found to be positively associated with AAA regardless of study types, however, confounding effect resulted from recruitment of poor control group may bias the estimation. Previous studies indicated a possible association between smoking status and AAA prevalence in COPD patients. For example, Pitoulias *et al*. suggested that the patients’ smoking status might affect the statistical outcome of risk estimation^23^. Axelrod *et al*. believed that smoking but not COPD determined the positive association between COPD and AAA, which was supported by two viewpoints: smoking is the primary risk factor of COPD and most patients with COPD have smoking history^24^. To confirm this assumption we made pooled estimation based on data adjusted for smoking status, and the result suggested that the positive association between AAA and COPD was independent of smoking status. Our estimation is consistent with Meijer *et al*.’s study which suggested that COPD was more prevalent in aneurysm patients than in controls (OR 3.0; 95% CI 1.6–5.5), and the association was independent of smoking stauts^25^. The above-mentioned findings suggested that COPD might be a risk factor for AAA regardless of patients’ smoking status.

The pooled adjusted data for short-term mortality showed that COPD was associated with increased risk of postoperative mortality. When categorized by type of treatment, our result suggested a significant association between COPD and short-term mortality in open surgery subgroup, but not in EVAR group. This difference might be attributed to the larger trauma of open repair. When categorized by type of AAA, our result suggested that the positive association between COPD and short-term mortality was stronger in rupture AAA subgroup than that in non-rupture AAA group. One of the possible explanation is the unstable hemodynamic status in patients with rupture AAA, and the COPD might aggravate the risk trend of short-term mortality.

Our result also showed that COPD is associated with increased short-term morbidity among AAA patients. These results were also supported by clinical based studies. For instance, several clinical studies confirmed that a significant proportion (9–40%) of pulmonary complications were developed in severe COPD patients undergoing abdominal surgery^26^. In addition, based on a retrospectively study involving 181 patients, COPD was found to be a high-risk factor and onset of postoperative respiratory failure in abdominal aortic surgery^27^. Similar to this study, Prinssen and colleagues found a significantly higher rate of pulmonary complications in the open repair group^28^. Moreover, Huber *et al*. analyzed the outcome of 16450 intact AAA underwent open repair by performing multivariate regression, and the result showed that COPD was positive associated with negative outcomes (adjusted OR 1.3; 95% CI 1.1–1.4). Unfortunately, these studies did not comprehensively analyze morbidities and evaluate the morbidity regarding different complications. Moreover, in evaluation of the long-term mortality, our study found that the patients with oxygen dependent COPD presented a 2-folder increased risk of AAA than those with medical COPD. This is also consistant with Van Laarhoven *et al*.’s finding which indicate that COPD appears to have a linear dose response to AAA, which meant that patients with more severe COPD (forced expiratory volume in the first second [FEV_1_]/vital capacity ratio <53% predicted) were more likely to have AAA than patients with less severe disease (19.3% vs 7.6%)^10^. However, in our review, different studies had different period of follow up, spanning from 1 year to 20 years, which might increase the discrepancy of outcome.

This meta-analysis had some inherent limitations. First, we did not control for confounding effect due to the development and changes in treatment, postoperative treatment protocols and patient selection criteria over time. When comparing outcomes of open versus endovascular repair in AAA patients with COPD from 1982 to 2015, there is a huge improvement during the last 10–15 years (compared to 25–30 years before). This improvement can be partially attributed to dramatic changes in preoperative evaluation, selections of treatment approach and postoperative treatment algorithms for AAA patients with COPD, especially considering the use of bronchodilators, epidural analgesia and changes in ICU protocols. Also, some AAA patients with severe comorbidities possibly had not been offered with any treatment (open repair or EVAR) due to the publication of EVAR trial 1^29^,^30^,^31^ and EVAR trial 2^32^ report. As a result, postoperative mortality and morbidity may have been decreased due to different patient selection criteria; therefore, mortality of either open repair or EVAR in COPD may have been differed over time. However, due to lack of such detailed information, we were unable to control for those potential confounding. Second, due to lack of well-conducted randomized trial, the validity of our study findings were compromised by limited confounder control. About 50% of the included studies were PBS, among which the heterogeneity can be resulted from differences in populations, definition of AAA and variable measurements, adjusted factors, and duration of follow-up. Additionally, the small number of studies limited our ability to compare the outcomes across subgroups. For example, EVAR subgroup and medical COPD subgroup only included 2 studies and oxygen dependent COPD subgroup included 3 studies, which might weaken the credibility of the results of sensitivity analysis. Finally, although the Egger test confirmed that most of pooled results had low publication bias, it is possible that we have missed some studies published in other languages rather than in English language.

## Conclusions

As there is an increasing prevalence of AAA in COPD patients, and the AAA postoperative mortality and morbidity rate is also higher among AAA patients with COPD compared with those without COPD, our study shows the epidemiological and clinical value to pay more attention for AAA prevention in COPD patients and make low-risk treatment for AAA patients with COPD. To better understand the association between COPD and AAA, as well as the rationale behind it, more well-conducted, large sample sized multicenter RCTs will be required to draw more valid conclusion.

## Additional Information

**How to cite this article**: Xiong, J. *et al*. Chronic obstructive pulmonary disease effect on the prevalence and postoperative outcome of abdominal aortic aneurysms: A meta-analysis. *Sci. Rep*. **6**, 25003; doi: 10.1038/srep25003 (2016).

## Supplementary Material

Supplementary Information

## Figures and Tables

**Table 1:** Abdominal aortic aneurysms (AAA) and chronic obstructive pulmonary disease (COPD): Prevalence and incidence studies.

Study (published year)	Study type	Male (%)	Age	Region	AAA (case/total)	No AAA (case/control)	Reported OR (95% CI)	P	Comments
Lederle *et al*. (1988)	PBS	100	≥60	US	1/18	7/183	NA	NA	
Smith *et al*. (1993)	PBS	100	≥65	UK	28/219	205/2378	NA	<0.05	
Simoni *et al*. (1995)	PBS	46	≥65	Italy	22/70	126/1504	NA	<0.001	
Lederle *et al*. (1997) (1)^¶^	PBS	97	≥50	US	NA	NA	1.04 (0.92–1.16)^*^	NA	AAA (3.0–3.9cm)
Lederle *et al*. (1997) (2)^¶^	PBS	97	≥50	US	NA	NA	1.28 (1.09–1.50)^*^	NA	AAA (≥4.0cm)
Lindholt *et al*. (1998)	PBS	100	≥65	Denmark	16/139	264/4265	2.05 (1.14–3.62)	0.01	
Lederle *et al*. (2000) (1)^§^	PBS	97	≥50	US	NA	NA	1.11 (0.95–1.30)^*^	NA	AAA (3.0–3.9cm)
Lederle *et al*. (2000) (2)^§^	PBS	97	≥50	US	NA	NA	1.08 (0.86–1.36)^*^	NA	AAA (≥4.0cm)
Shteinberg *et al*. (2000)	CCS	88	≥43	Israel	28/82	19/73	NA	NS	AAA vs. PAOD
Petersen *et al*. (2002)	CCS	70	≥50	Sweden	2/10	3/30	NA	NA	AAA vs (AOD + SCS + NAM)
Barba *et al*. (2005)	CCS	93.7	NA	Spain	40/151	184/1015	NA	0.015	AAA vs. PAOD
Fowkes *et al*. (2006)	CCS	72	74^m^	UK	87/89	94/98	NA	NA	AAA vs. No
Yaghoubian *et al*. (2006)	CCS	91	74^m^	US	14/100	1/100	16.1 (2.08–125.1)	0.0005	AAA vs. No
Iribarren *et al*. (2007)	PS	45	>18	US	NA	NA	1.25 (0.94–1.65)^*^	NA	AAA/No AAA: 605/104208
Koksal *et al*. (2007)	CCS	NA	≥47	Turkey	7/40	9/40	NA	NA	AAA vs. AOD
Ito *et al*. (2008)	CCS	78.7	73^m^	Japan	63/211	20/132	2.38 (1.28–4.26); 2.05 (1.11–3.89)^*^	0.002; 0.025^*^	AAA vs. TAA
Lederle *et al*. (2008)	PS	0	≥50	US	22/184	5657/161624	1.44 (0.88–2.37)^*^	NA	
Svensjo *et al*. (2011)	PBS	100	65	Sweden	21/233	920/14378	1.2 (0.8–1.9)^*^	0.44^*^	
Duncan *et al*. (2012)	PS	100	≥65	UK	73/414	623/7063	3.13 (2.28–4.30);1.98 (1.37–2.86)^*^	NA	
Pitoulias *et al*. (2012)	CCS	88.2	≥45	Greece	35/110	10/60	0.43 (0.20–0.94);0.65 (0.27–1.58)^*^	0.035 0.34^*^	AAA vs. AOD
Saracini *et al*. (2012)	CCS	87.7	≥40	Italy	311/423	66/423	NA	NA	AAA vs. No
Svensjo *et al*. (2013)	PBS	0	70	Sweden	3/19	447/5120	1.96 (0.57–6.75)	0.277	
Chun *et al*. (2014)	PBS	99.6	≥50	US	122/469	647/5673	1.75 (1.41–2.18)	<0.001	

PBS: population based screening; CCS: case control study; PS: prospective study; NA: not available, NS: not significant; AOD: aortic occlusive disease; SCS: plaque ulceration; NAM: no similar specific manifestations of atherosclerotic disease; PAOD: peripheral artery occlusive disease; TAA: thoracic aorta aneurysm; ^¶^: accumulated data in the two studies (AAA/No AAA: 3366/70085); §: accumulated data in the two studies (AAA/No AAA: 1917/50828); ^m^ mean; ^*^adjusted data available.

Reference in supplemental files.

**Table 2:** Postoperative outcome in chronic obstructive pulmonary disease (COPD) and non-COPD patients with abdominal aortic aneurysms (AAA).

Study (published year)	Study type	Male (%)	Age	Region	AAA	30-d mortality	Long term mortality	30-d morbidity	Long-term morbidity	Comment
Crawford *et al*. (1986)	PBS	85.1	28–85	US	101 elective open COPD: 34 Non-COPD: 67	30-d 1 (2.9%) 7 (10.4%) (P = 0.2611)		30-d Dialysis 2 (5.9%) 5 (7.5%) (P = 1.000)		
Katz *et al*. (1994)	PBS	82	≥50	US	8185 open COPD: 1792 Non-COPD: 6393	In-hospital 142 (7.9%) 473 (7.4%) (P = 0.6)				Elective: 8185 Rupture: 1829
Koskas *et al*. (1997)	PS	89.9	42–92	France	158 elective open COPD: 51 Non-COPD: 107		5.3-y (P < 0.01)			
Eskandari *et al*. (oxygen)	CCS	76.9	72^m^	US	65 elective open COPD: 14 Non-COPD: 51			In-hospital 4 (28.6%) 18 (35.3%)		Oxygen dependent COPD
Cuypers *et al*. (2000)	PBS	91.8	70^m^	Netherland	1871 elective EVAR COPD: 683 Non-COPD: 1188				1.5-y Conversion 27 (4.0%) 22 (1.9%) OR 2.22 (1.12–4.37)^*^	
Axelrod *et al*. (2001)	PBS	100	69^m^	US						
Rupture					52 open rupture COPD: 20 Non-COPD: 32	30-d 8 (40.0%) 2 (6.3%)				
No-rupture					1001 elective open COPD: 244 Non-COPD: 757	30-d 9 (3.7%) 28 (3.7%) OR 1.1(P = 0.81)^*^		30-d OR 2.3 (P = 0.07)^*^In ventilation >96 h		
Huber *et al*. (2001)	PBS	79.7	72^m^	US	16450 elective open	In-hospital OR 1.0; 95%CI 0.9–1.3 (P = 0.67)^*^		In-hospital OR 1.3; 95% CI 1.1–1.4 (P < 0.0001)^*^		
Biancari *et al*. (2002)	PBS	90.4	66^m^	Finland	208 EVAR COPD: 54 Non-COPD: 154		15-y (P = 0.001) RR 1.76, 95% CI 1.22–2.44 (P = 0.002)^*^			Elective: 167; Emergency for no rupture: 9; Emergency for rupture: 32
Piper *et al*. (2003)	PBS	68.4	72^m^	US	147 open rupture COPD: 35 Non-COPD: 112	In-hospital 9 (25.7%) 42 (37.5%)				
Tassiopoulos *et al*. (2004)	PBS	81.7	71^m^	US	115 elective open COPD: 22 Non-COPD: 93			In-hospital Ventilator time (P = 0.12) ICU stay (P = 0.015) Postoperative ileus (NS) Hospital stay (P = 0.03)		
Hertzer *et al*. (2005)	PBS	86	≥47	US	855 elective open COPD: 53 Non-COPD: 802	30-d OR 3.8; 95% CI 1.2–11.6 (P = 0.036) OR 5.1; 95% CI 1.6–16.5 (P = 0.0006)^*^	15-y HR 1.55; 95% CI 1.11–2.17 (P = 0.016) HR 1.6 95% CI 1.1–2.2 (P = 0.012)^*^			HR: any death (postoperative and late death)
Hua *et al*. (2005)	PS	81.8	72^m^	US	1042 elective EVAR and open COPD: 221 Non-COPD: 821	30-d OR 1.67; 95%CI 0.81–3.44 (P = 0.17)		30-d OR 1.42; 95CI% 1.03–1.94 (P = 0.03) OR 1.32; 95%CI 0.91–1.91 (P = 0.15)^*^		EVAR: 460 Open: 582
Schouten *et al*. (2006)	PBS	86	70^m^	Netherland	500 elective open COPD: 31 Non-COPD: 469	30-d OR 1.83; 95% CI 0.86–3.87 (P = 0.12) OR 1.50; 95% CI 0.63–3.58 (P = 0.36)^*^		30-d OR 1.91; 95% CI 0.93–3.93(P = 0.08) OR 1.81; 0.85–3.89 (P = 0.13)^*^		
Zarins *et al*. (2006)	PS	88.3	74^m^	US	923 elective EVAR COPD: 90 Non-COPD: 833		5-y HR 1.84 (P < 0.0001)^*^AAA related death HR 1.75 (P = 0.18)^*^		5-y AAA rupture HR 0.987 (P = 0.98)^*^Conversion HR 1.07 (P = 0.18)^*^	
Anain *et al*. (2007)	PBS	75	57–89	US	40 EVAR and open rupture COPD: 17 Non-COPD: 23	30-d 5 (29.4%) 4 (17.4%)				EVAR: 30 Open: 10
Bonardelli *et al*. (2007)	PBS	93.5	NA	Italy	1111 elective open COPD: 428 Non-COPD: 683	30-d 14 (3.3%) 16 (2.3%) (P = 0.35)	5-y (P < 0.001) (P = 0.014)^*^			
Berge *et al*. (2008)	PBS	82.9	71^m^	US	1041 EVAR and open COPD: 144 Non-COPD: 897	30-d Open OR 1.2; 95% CI 0.7–2.1 (P = 0.48)^*^	20-y OR 1.5; 95% CI 1.17–1.92 (P = 0.001)^*^			EVAR: 136 Open rupture: 299; Open no rupture: 606
Botha *et al*. (2008)	PBS	80.6	76^m^	Australia	62 open rupture COPD: 17 Non-COPD: 45	In-hospital 8 (47.1%) 12 (26.7%) OR 6.7; 95% CI 1.1–41.3 (P = 0.04)^*^				
Park *et al*. (2008)	CCS	88.6	76^m^	US	342 elective EVAR COPD: 137 Non-COPD: 205	In-hospital 3 (2.2%) 4 (2.0%) (P = 1.0)		In-hospital 18 (40.1%) 18 (43.9%)		Long-term mortality: COPD (CHF: 3; MI:1;; RC: 14); Non-COPD (CHF: 2; MI: 2; RC: 14)
Abedi *et al*. (2009)	PBS	82.3	74^m^	US	3662 elective EVAR COPD: 700 Non-COPD: 2962			30-d OR 1.31 (P < 0.05)^*^in postoperative transfusion 4u. w/in 72 h of procedure OR 2.28 (P < 0.05)^*^		
Antonello *et al*. (2009)	PBS	79.6	47–91	Italy	103 open rupture COPD: 35 Non-COPD: 68	30-d 15 (42.9%) 15 (22.1%) (P = 0.028)				
Beck *et al*. (2009)	PS	71	74^m^	US						
Open					748 elective open COPD: 269 Non-COPD: 479		1-y P = 0.002 HR 3.6; 95% CI 1.9–7.0^*^			
EVAR					639 elective EVAR COPD: 249 Non-COPD: 390		1-y P = 0.007			
Holst *et al*. (2009)	PBS	86	76^m^	Sweden	90 EVAR rupture COPD: 28 Non-COPD: 62	30-d RR 4.3; 95%CI 1.7–11.0 (P = 0.003)^*^				
Mastracci *et al*. (2010)	PS	88	75^m^	US	412 elective EVAR COPD: 155 Non-COPD: 257		6-y 106 (68.4%) 136 (52.9%) HR1.6; 95% CI 1.3–2.1 (P = 0.002) HR 1.6; 95% CI 1.2–2.2 (P = 0.001)^*^			
Geisbüsch *et al*. (2011)	PBS	85	>80	US, Germany	299 elective EVAR COPD: 90 Non-COPD: 209		6.8-y HR 1.56; 95% CI 1.03–2.35 (P = 0.032)^*^			
Twine *et al*. (2011)	PBS	92.7	55–88	UK	178 elective EVAR COPD: 33 Non-COPD: 145		3-y HR 0.42; 95% CI 0.25–0.72 (P = 0.002)			
Wisniowski *et al*. (2011)	PBS	87.3	73^m^	Australia	197 elective EVAR COPD: 39 Non-COPD: 158		3-y (P = 0.027) (P = 0.035)^*^			
Gupta *et al*. (2012)	PBS	72.5	66–78	US	598 elective open COPD: 122 Non-COPD: 476	30-d 11 (9.0%) 16 (3.4%) OR 3.17; 95% CI 1.39–7.25^*^		30-d Major complication: 48 (39.3%) 132 (27.7%) (P = 0.01)		
Ohrlander *et al*. (2012)	PS	86	53–89	Sweden	233 elective EVAR COPD: 95 Non-COPD: 138		5-y 38 (40.0%) 39 (28.3%) (P = 0.058)			Long-term mortality COPD (grade ≥2) (P = 0.040) COPD (grade ≥3) (P = 0.016) PaO2 < 8.0 kPa or COPD, grade ≥3 (P = 0.005) HR 2.06; 95% CI 1.24–3.42 (P = 0.005)^*^
De Martino *et al*. (2013)	PBS	79.3	67–82	US	2367 elective EVAR and open					
Untreated					1897 elective EVAR and open COPD: 336 Non-COPD: 1562		5-y OR 1; 95% CI 0.7–1.3^*^			EVAR: 1303 Open: 594 Untreated COPD
Medical					1936 elective EVAR and open COPD: 374 Non-COPD: 1562		5-y OR 1.3; 95% CI 1.0–1.8^*^			EVAR: 1355 Open: 579 Medication COPD
Oxygen					1659 elective EVAR and open COPD: 97 Non-COPD: 1562		5-y OR 3.0; 95% CI 2.0–4.5^*^			EVAR: 1165 Open: 493 Oxygen dependent COPD
Stone *et al*. (2013)	PS	78.1	72^m^	US						
Medical					3320 elective EVAR and open COPD: 1082 Non-COPD: 2238		5-y HR 1.22; 95% CI 1–1.5 (P = 0.02)^*^			EVAR: 2043 Open: 1412 Medical COPD
Oxygen					2373 elective EVAR and open COPD: 135 Non-COPD: 2238	In-hospital OR 2.02; 95% CI 1.0–4.0 (P = 0.04)^*^	5-y HR 3.02; 95%C 2.2–4.1 (P < 0.001)^*^			Oxygen dependent COPD
Raux *et al*. (2014)	CCS	76.6%	73^m^	US and France	563 elective FEVAR and open			30-d OR 3.3; 95%CI 1.7–6.7 (P = 0.0008)^*^		

PBS: population based screening; CCS: case control study; PS: prospective study; COPD: chronic obstructive pulmonary disease; EVAR: endovascular aortic repair; OR: odd ratio; RR: relative risk; HR: hazard rate; CI: confidence interval; SDD: same day discharge; POD: postoperative discharge; CHF: congestive heart failure; MI: myocardial infarction; RC: respiratory complications; ICU: intensive care unit; NS: not significant; NA: not available; O_2_: oxygen; ^m^ mean; ^*^adjusted data available.

Reference in supplemental files.
