# Supplementary material for: Chronic obstructive pulmonary disease effect on the prevalence and postoperative outcome of abdominal aortic aneurysms: A meta-analysis
Source: Sci Rep. 2016 Apr 26;6:25003. doi: 10.1038/srep25003 (PMC4845024; doi:10.1038/srep25003)
Supplement: Supplementary Information [file srep25003-s1.doc]

Chronic obstructive pulmonary disease effect on the prevalence and postoperative outcome of abdominal aortic aneurysms: A meta-analysis

Jiang Xiong, MD, PhD^1^, Zhongyin Wu, MD^1, 2^, Chen Chen, DrPH^3^, Wei Guo, MD^1^*

From the ^1^ Department of Vascular and Endovascular Surgery, Chinese PLA General Hospital, Beijing, P.R. China; ^2^ Department of General Surgery, Affiliated Hospital of Chengde Medical College, Chengde, Hebei, P.R. China; ^3^ Department of Health Policy and Management, Jiann-Ping Hsu College of Public Health, Georgia Southern University, Statesboro, GA, USA

*Correspondence author: Wei Guo, Department of Vascular and Endovascular Surgery, Chinese PLA General Hospital, 28 Fuxing Rd, Haidian District, Beijing, 100853, China;

Tel: 86-10-66938349; Fax: 86-10-68176994; E-mail: pla301dml@vip.sina.com

Key words: abdominal aortic aneurysm, chronic obstructive pulmonary disease, prevalence, postoperative outcome, meta-analysis

Online Table 1A. Methodological quality of included studies by NOS. Cohort studies

|  |  | **Methodology Quality assessment** | | | | | | | | |
| --- | --- | --- | --- | --- | --- | --- | --- | --- | --- | --- |
| **Author, published year** | **setting** | **Selection** | | | | **Comparability** | **Outcome** | | |  |
|  |  | **Representativeness** | **Selection**  **nonexposed** | **Ascertainment**  **Exposure** | **Absence Outcome** | **Comparability** | **Assessment**  **outcome** | **Adequate**  **Duration f-up°** | **Adequate**  **f-up°** | **TOT**  **Stars** |
| Lederle et al. (1997) | Large screening | C (0) | A (1) | C (0) | A (1) | 0 | A (1) | N.A. | N.A. | 3 |
| Lindholt et al. (1998) | Large screening | A (1) | A (1) | B (1) | A (1) | 0 | B (1) | N.A. | N.A. | 5 |
| Lederle et al. (2000) | Large screening | C (0) | A (1) | C (0) | A (1) | 0 | A (1) | N.A. | N.A. | 3 |
| Svensjo et al. (2011) | Large screening | A (1) | A (1) | C (0) | A (1) | 0 | A (1) | N.A. | N.A. | 4 |
| Iribarren et al. (2007) | Prospective | A (1) | A (1) | B (1) | A (1) | 0 | A (1) | A (1) | B (1) | 7 |
| Lederle et al. (2008) | Prospective | B (1) | A (1) | B (1) | B (0) | 0 | A (1) | A (1) | B (1) | 6 |
| Duncan et al. (2012) | Prospective | A (1) | A (1) | B (1) | A (1) | 0 | A (1) | A (1) | B (1) | 7 |
| Lederle et al. (1988) | Screening | B (1) | A (1) | B (1) | A (1) | 0 | A (1) | N.A. | N.A. | 5 |
| Smith et al. (1993) | Screening | B (1) | A (1) | B (1) | A (1) | 0 | A (1) | N.A. | N.A. | 5 |
| Simoni et al. (1995) | Screening | B (1) | A (1) | C (0) | A (1) | 0 | A (1) | N.A. | N.A. | 4 |
| Svensjo et al. (2013) | Screening | B (1) | A (1) | C (0) | A (1) | 0 | A (1) | N.A. | N.A. | 4 |
| Chun et al. (2014) | Screening | B (1) | A (1) | B (1) | A (1) | 0 | A (1) | N.A. | N.A. | 5 |
| Crawford et al. (1986) | Clinical data | B (1) | A (1) | B (1) | A (1) | 0 | A (1) | B (0) | C (0) | 5 |
| Katz et al. (1994) | Clinical data | A (1) | A (1) | B (1) | A (1) | 0 | A (1) | B (0) | D (0) | 5 |
| Cuypers et al. (2000) | Clinical data | B (1) | A (1) | B (1) | A (1) | 0 | A (1) | B (0) | D (0) | 5 |
| Axelrod et al. (2001) | Clinical data | B (1) | A (1) | B (1) | A (1) | 0 | A (1) | B (0) | D (0) | 5 |
| Huber et al. (2001) | Clinical data | B (1) | A (1) | B (1) | A (1) | 0 | A (1) | B (0) | D (0) | 5 |
| Biancari et al. (2002) | Clinical data | A (1) | A (1) | B (1) | A (1) | 0 | A (1) | A (1) | B (1) | 7 |
| Piper et al. (2003) | Clinical data | A (1) | A (1) | B (1) | A (1) | 0 | A (1) | B (0) | D (0) | 5 |
| Tassiopoulos et al. (2004) | Clinical data | A (1) | A (1) | B (1) | A (1) | 0 | A (1) | B (0) | D (0) | 5 |
| Hertzer et al. (2005) | Clinical data | A (1) | A (1) | A (1) | A (1) | 0 | A (1) | A (1) | C (0) | 6 |
| Hua et al. (2005) | Clinical data | A (1) | A (1) | B (1) | A (1) | 0 | A (1) | B (0) | D (0) | 5 |
| Schouten et al. (2006) | Clinical data | A (1) | A (1) | A (1) | A (1) | 0 | A (1) | B (0) | D (0) | 5 |
| Zarins et al. (2006) | Clinical data | B (1) | A (1) | B (1) | A (1) | 0 | A (1) | A (1) | B (1) | 7 |
| Anain et al. (2007) | Clinical data | A (1) | A (1) | A (1) | A (1) | 0 | A (1) | B (0) | D (0) | 5 |
| Bonardelli et al. (2007) | Clinical data | A (1) | A (1) | B (1) | A (1) | 0 | A (1) | A (1) | B (1) | 7 |
| Koskas et al. (2007) | Clinical data | A (1) | A (1) | B (1) | A (1) | 0 | A (1) | B (0) | C (0) | 5 |
| Berge et al. (2008) | Clinical data | A (1) | A (1) | B (1) | A (1) | 0 | A (1) | A (1) | B (1) | 7 |
| Botha et al. (2008) | Clinical data | A (1) | A (1) | B (1) | A (1) | 0 | A (1) | B (0) | D (0) | 5 |
| Park et al. (2008) | Clinical data | B (1) | A (1) | A (1) | A (1) | 0 | A (1) | B (0) | D (0) | 5 |
| Abedi et al. (2009) | Clinical data | B (1) | A (1) | A (1) | A (1) | 0 | A (1) | B (0) | D (0) | 5 |
| Antonello et al. (2009) | Clinical data | A (1) | A (1) | B (1) | A (1) | 0 | A (1) | B (0) | D (0) | 5 |
| Beck et al. (2009) | Clinical data | C (0) | A (1) | B (1) | A (1) | 0 | A (1) | B (0) | D (0) | 4 |
| Holst et al. (2009) | Clinical data | B (1) | A (1) | B (1) | A (1) | 0 | A (1) | B (0) | D (0) | 5 |
| Mastracci et al. (2010) | Clinical data | B (1) | A (1) | B (1) | A (1) | 0 | A (1) | A (1) | D (0) | 6 |
| Geisbusch et al. (2011) | Clinical data | B (1) | A (1) | B (1) | A (1) | 0 | A (1) | A (1) | B (1) | 7 |
| Twine et al. (2011) | Clinical data | B (1) | A (1) | B (1) | A (1) | 0 | A (1) | B (0) | D (0) | 5 |
| Wisniowski et al. (2011) | Clinical data | B (1) | A (1) | A (1) | A (1) | 0 | A (1) | B (0) | B (1) | 6 |
| Gupta et al. (2012) | Clinical data | B (1) | A (1) | A (1) | A (1) | 0 | A (1) | B (0) | D (0) | 5 |
| Ohrlander et al. (2012) | Clinical data | B (1) | A (1) | A (1) | A (1) | 0 | A (1) | A (1) | D (0) | 6 |
| De Martino et al. (2013) | Clinical data | B (1) | A (1) | B (1) | A (1) | 0 | A (1) | A (1) | D (0) | 6 |
| Stone et al. (2013) | Clinical data | A (1) | A (1) | B (1) | A (1) | 0 | A (1) | A (1) | C (0) | 6 |

NOS: Newcastle-Ottawa Scale

Online Table 1B. Methodological quality of included studies by NOS. Case-control studies

|  |  | **Methodology Quality assessment** | | | | | | | | |
| --- | --- | --- | --- | --- | --- | --- | --- | --- | --- | --- |
| **Author, published year** | **setting** | **Selection** | | | | **Comparability** | **Exposure** | | | **TOT stars** |
|  |  | **Case definition** | **Representativeness** | **Control selection** | **Control definition** | **Comparability** | **Ascertainment** | **Method case/controls** | **No-response** |  |
| Eskandari et al. (1999) | Case-control | A (1) | B (0) | B (0) | A (1) | 0 | A (1) | A (1) | C (0) | 4 |
| Shteinberg et al. (2000) | Case-control | A (1) | B (0) | B (0) | A (1) | 0 | A (1) | A (1) | C (0) | 4 |
| Petersen et al. (2002) | Case-control | A (1) | B (0) | B (0) | A (1) | B (1) | A (1) | A (1) | C (0) | 5 |
| Barba et al. (2005) | Case-control | A (1) | A (1) | B (0) | A (1) | 0 | A (1) | A (1) | C (0) | 5 |
| Fowkes et al. (2006) | Case-control | A (1) | A (1) | C (0) | A (1) | A (1) | A (1) | A (1) | C (0) | 6 |
| Yaghoubian et al. (2006) | Case-control | A (1) | A (1) | B (0) | A (1) | 0 | A (1) | A (1) | C (0) | 5 |
| Koksal et al. (2007) | Case-control | A (1) | B (0) | B (0) | A (1) | 0 | A (1) | A (1) | C (0) | 4 |
| Ito et al. (2008) | Case-control | A (1) | B (0) | B (0) | A (1) | A (1) | A (1) | A (1) | C (0) | 5 |
| Pitoulias et al. (2012) | Case-control | A (1) | B (0) | B (0) | A (1) | A (1) | A (1) | A (1) | C (0) | 5 |
| Saracini et al. (2012) | Case-control | A (1) | B (0) | C (0) | A (1) | A (1) | A (1) | B (0) | C (0) | 4 |
| Raux et al. (2014) | Case-control | A (1) | B (0) | B (0) | A (1) | A (1) | A (1) | A (1) | C (0) | 5 |

NOS: Newcastle-Ottawa Scale


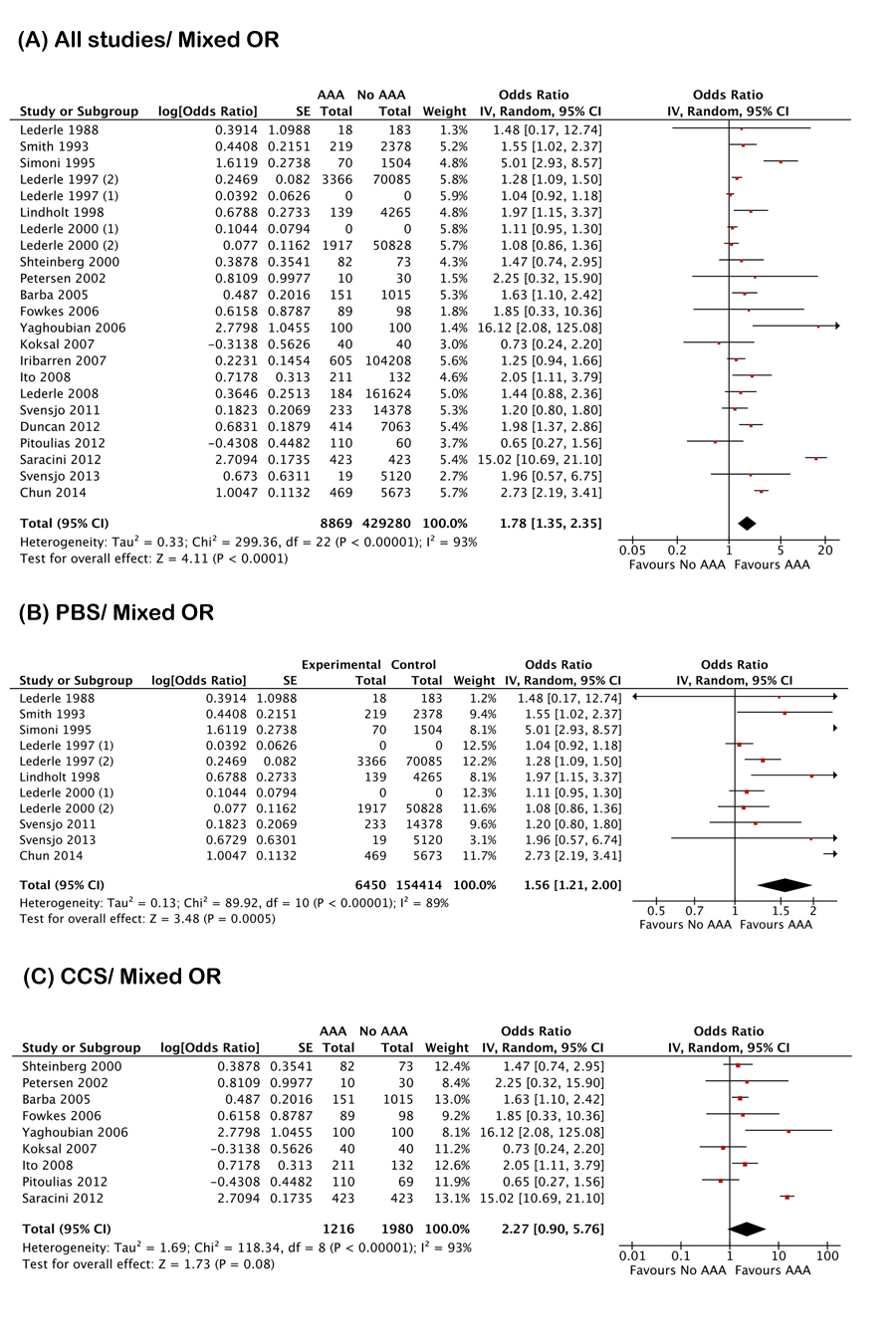


Online figure 1. The association between chronic obstructive pulmonary disease and abdominal aortic aneurysm: Pooled mixed odd ratio. (A) All studies. (B) Population based screenings. (C) Case-control studies.


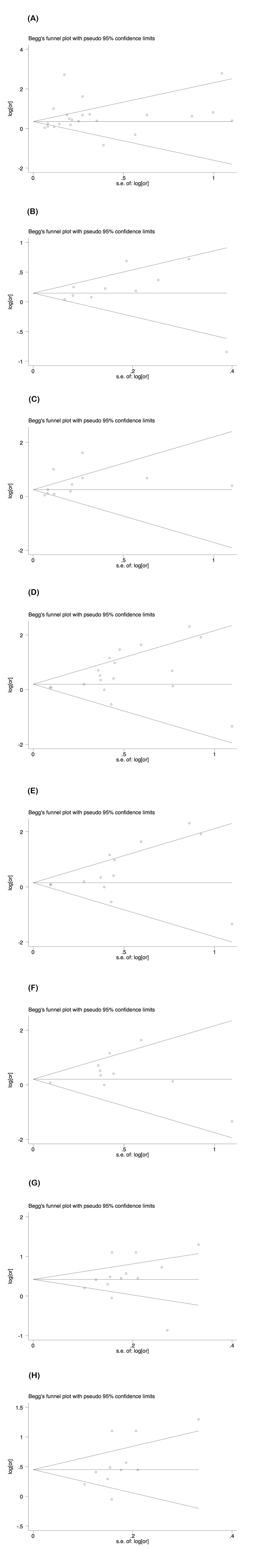

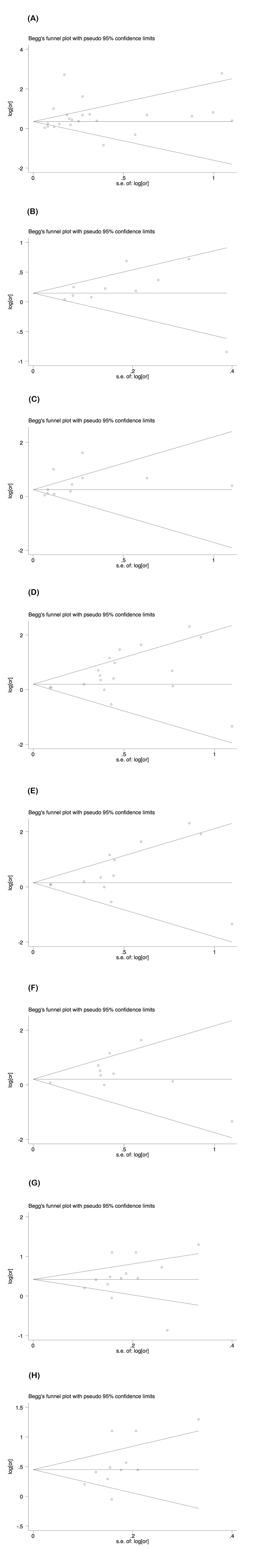


Online figure 2. Funnel plots of log of odd ratio to explore publication bias. (A) Prevalence of abdominal aortic aneurysm (AAA) in chronic obstructive pulmonary disease (COPD) using mixed data. (B) Prevalence of AAA in COPD using adjusted data. (C) Prevalence of AAA in COPD using mixed data: population-based screening. (D) Operative (30-day/in-hospital) mortality using mixed data. (E) Operative (30-day/in-hospital) mortality using mixed data: Open repair. (F) Operative (30-day/in-hospital) mortality using mixed data: Elective repair. (G) Operative (long-term) mortality using mixed data. (H) Operative (long-term) mortality using adjusted data.


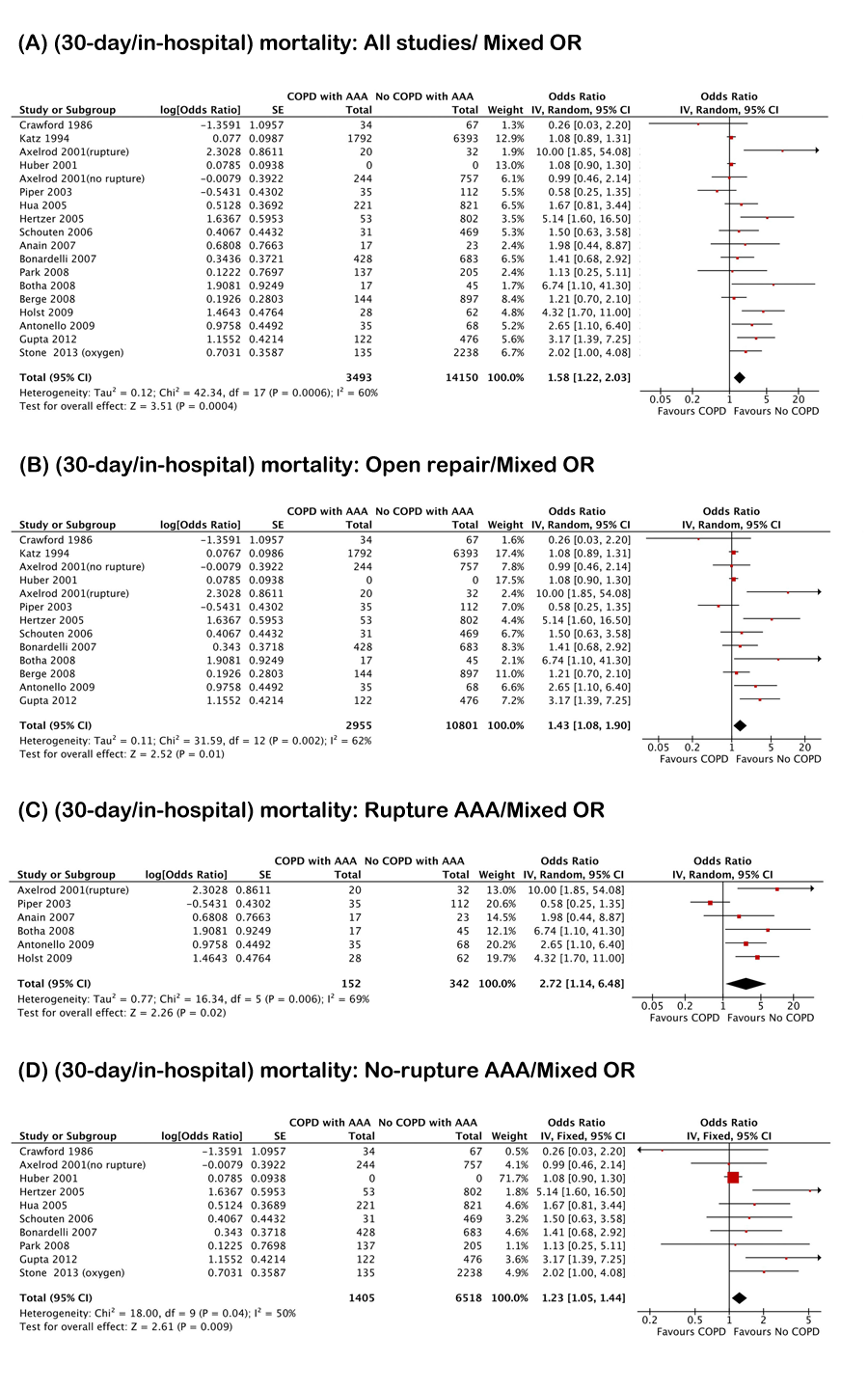


Online figure 3. Cumulative operative mortality in abdominal aortic aneurysm (AAA) patients with chronic obstructive pulmonary disease: Pooled mixed odd ratio. (A) All studies. (B) Studies with AAA accepted open repair. (C) Studies with rupture AAA. (D) Studies with no rupture AAA.


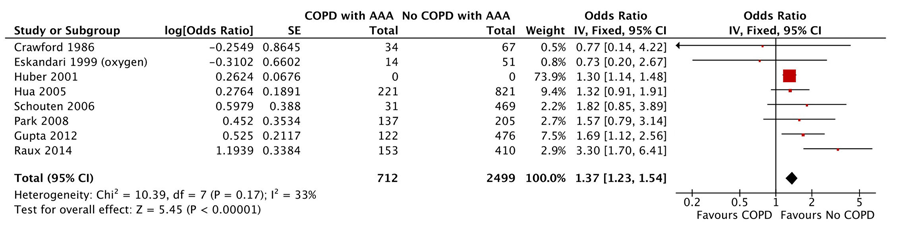


Online figure 4. Cumulative operative morbidity in abdominal aortic aneurysm patients with chronic obstructive pulmonary disease: Pooled mixed odd ratio.


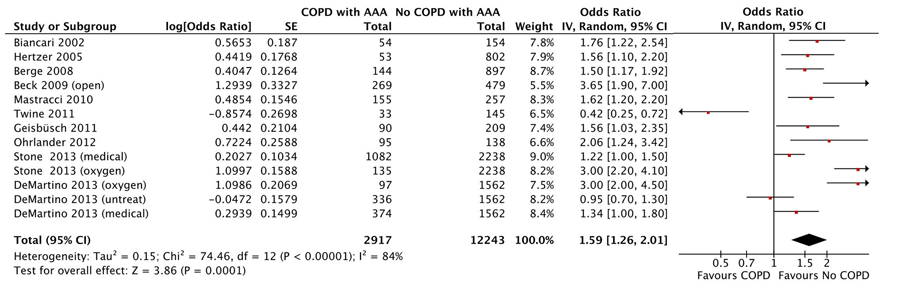


Online figure 5. Cumulative long-term mortality in abdominal aortic aneurysm patients with chronic obstructive pulmonary disease: Pooled mixed odd ratio.

Table 1

Reference
